# Risk Factors Associated with the Seroprevalence of Leptospirosis in Small Ruminants from a Semi-Arid Region of Mexico

**DOI:** 10.3390/pathogens14040344

**Published:** 2025-04-03

**Authors:** Jesús Francisco Chávez-Sánchez, Lucio Galaviz-Silva, Zinnia Judith Molina-Garza, Pablo Zapata-Benavides, Sibilina Cedillo-Rosales, Joel Horacio Elizondo-Luévano, Miroslava Kačániová, Ramiro Ávalos-Ramírez

**Affiliations:** 1 Universidad Autónoma de Nuevo León, Facultad de Medicina Veterinaria y Zootecnia, Departamento de Virología, Cuerpo Académico de Epidemiología Veterinaria, Campus Ciencias Agropecuarias, Mariano Escobedo, Nuevo León C.P. 66054, Mexico; jesus.chavezsnc@uanl.edu.mx (J.F.C.-S.); sibilina.cedillors@uanl.edu.mx (S.C.-R.); 2 Universidad Autónoma de Nuevo León, Facultad de Ciencias Biológicas, Laboratorio de Patología Molecular y Experimental, Ave. Universidad, S/N, Ciudad Universitaria, San Nicolas de los Garza, Nuevo León C.P. 66455, Mexico; zinnia.molinagr@uanl.edu.mx; 3 Universidad Autónoma de Nuevo León, Facultad de Ciencias Biológicas, Laboratorio de Inmunología y Virología, Ave. Universidad, S/N, Ciudad Universitaria, San Nicolas de los Garza, Nuevo León C.P. 66455, Mexico; pablo.zapatabn@uanl.edu.mx; 4 Facultad de Farmacia, Instituto de Investigación Biomédica de Salamanca, Universidad de Salamanca, Campus Miguel de Unamuno s/n, 37007 Salamanca, Spain; jelizondo@usal.es; 5 Biomolecular Innovation Group, Laboratorio de Ciencias Naturales, Facultad de Agronomía, Universidad Autónoma de Nuevo León, Campus Ciencias Agropecuarias, Mariano Escobedo, Nuevo León C.P. 66054, Mexico; 6 Institute of Horticulture, Faculty of Horticulture and Landscape Engineering, Slovak University of Agriculture, Tr. A. Hlinku 2, 94976 Nitra, Slovakia; miroslava.kacaniova@gmail.com; 7 School of Medical & Health Sciences, University of Economics and Human Sciences in Warsaw, Okopowa 59, 01 043 Warszawa, Poland

**Keywords:** *Leptospira*, goats, sheep, public health, neglected zoonoses, odds ratios, spirochetes, biosecurity, MAT

## Abstract

Leptospirosis is one of the world’s major neglected tropical zoonotic diseases (NTZDs), implicated in animal health and welfare with economic consequences for livestock production. This study aims to estimate the seroprevalence of *Leptospira* spp. and identify potential risk factors in small ruminant herds. This epidemiological cross-sectional study was conducted in Nuevo León, a semi-arid region of Mexico. A total of 389 blood samples from goats and 385 from sheep older than eight months were randomly collected from 128 herds. Anti-*Leptospira* antibodies were detected using the microscopic agglutination test (MAT), and univariate and multivariate logistic regression analyses were performed to determine their association with leptospirosis infection. The overall prevalence was 13.5% (105/774), with 14.4% (56/389) in goats and 12.7% (49/385) in sheep. Sejroe was the most predominant serogroup. The main risk factors in sheep were contact with domestic cattle, ≥100 animals per herd, congenital abnormalities, contact with feral pigs, meat production system, absence of veterinary care, and abortions with odds ratios (OR) between 1.7 and 4.1. In goats, the main risk factors included lack of quarantine measures, contact with feral pigs, absence of veterinary care, and abortions where the OR ranged from 1.7 to 3.3. These findings indicate that *Leptospira* spp. is present in small ruminant herds. This is the first study aimed at understanding leptospirosis epidemiology in the northeastern region of Mexico, as goats and sheep may act as potential reservoirs. Continuous monitoring of *Leptospira* infections is imperative, as well as developing educational initiatives for farmers to implement biosecurity and prevention measures to prevent infections within herds and protect public health.

## 1. Introduction

Leptospirosis affects many animal species, both domestic and wild; it is a globally neglected zoonotic disease caused by spirochetes belonging to the genus *Leptospira* [1]. There are over 250 pathogenic serovars [1,2], since *Leptospira* species exhibit significant antigenic differences due to multiple variations in their membrane lipopolysaccharide, leading to their classification into 25 serogroups [3,4]. Consequently, there is no direct correlation between the genetic classification of the species and their antigenic classification into serogroups and serovars [5]. Additionally, different *Leptospira* serovars demonstrate varying degrees of adaptability to different animal species. In some animal hosts, adapted serovars may cause chronic disease, establishing infections in the reproductive and renal tracts, leading to prolonged bacterial shedding through the genitourinary system [6,7].

In small ruminants, leptospirosis causes chronic reproductive disorders such as abortions, mummifications, reduced milk production, stillbirths, and perinatal mortality [8]. These issues translate into significant economic losses, particularly for small-scale farmers whose primary income depends on goat and sheep farming [9]. To mitigate economic losses, vaccination with regionally prevalent serogroups is recommended [10]. However, in Mexico, the immunization of small ruminants is not commonly practiced. Other factors that increase the probability of leptospirosis infection in small ruminants include coexistence with other domestic and wild animals, contact with contaminated abortions or placentas from infected animals, and lack of biosecurity measures such as veterinary assistance or quarantine protocols [11,12].

In northeastern Mexico, goat and sheep populations, along with other ruminants such as cattle and deer, coexist and interact with various domestic and wild animals [13,14]. This coexistence increases the risk of pathogen dissemination within the ecological community [15]. The northeastern region of Mexico is characterized by extensive arid and semi-arid zones, where most small ruminant herds operate under a family-based production system [16,17]. Goat and sheep production in these areas, particularly in the northeast part of the country, is an important social and agro-economic activity. The state of Nuevo León, primarily located in Mexico’s semi-arid region, has a livestock census of over 500,000 small ruminants, most of which are raised in family subsistence farms producing traditional artisanal cheeses, burnt milk caramel, and meats such as goatling and lamb [17].

In this semi-arid rural region, health disorders in goats and sheep are often caused by adverse climatic conditions that lead to constant physiological stress in association with viral and parasitic infections [14,18]. Leptospirosis is recognized as a complex disease with multiple transmission routes, a broad host range, a high number of infectious serovars, multifaceted clinical manifestations, and challenges in detection [19,20]. While leptospirosis is found worldwide, it is relatively more significant in tropical and subtropical regions where environmental conditions favor the survival of pathogenic *Leptospira* in surface water and moist soils [21]. However, disease and bacterial presence have also been reported in desert and semi-desert regions, where survival and transmission dynamics among animals may differ from those in tropical and temperate regions [12,22].

Although most reports of leptospirosis in small ruminants describe asymptomatic infections and highlight their role in the epidemiology of the disease through bacterial shedding in urine [23], pathogenic *Leptospira* can also clinically affect small ruminants [8]. Recent studies on leptospirosis in small ruminants in Mexico are limited to tropical regions, with seroprevalence rates ranging from 53.8% to 71.1% [11,24,25]. Unfortunately, no information exists regarding leptospirosis in semi-arid regions of Mexico. Therefore, the objective of this study was to determine the seroprevalence and to correlate seropositivity with potential risk factors in small ruminant herds in a semi-arid region of northeastern Mexico.

## 2. Materials and Methods

### 2.1. Study Area

This study was conducted in the semi-arid region of Nuevo León, Mexico, between May 2021 and September 2022. Nuevo León is a state in northeastern Mexico, located between 23°06′ N and 27°50′ N latitude, and 98°17′ W and 101°07′ W longitude. The average daytime temperature is 20 °C, with an annual precipitation of 650 mm and a relative humidity of 70%. The region covers a total area of 64,801.94 km^2^ and is characterized by an arid climate, except for areas within the Sierra Madre Oriental, which have a temperate subhumid climate [26].

### 2.2. Study Design and Sample Collection

A cross-sectional observational study was conducted on randomly selected goat and sheep herds among willing owners in the region. Population data for each species were obtained from the staff of the Agricultural and Fisheries Information Service (SIAP), a decentralized agency of the Secretariat of Agriculture and Rural Development (SADER, https://www.gob.mx/siap/, accessed on 12 March 2025), in Monterrey, Nuevo León.

Sample size calculations were based on the regional goat and sheep populations (n = 413,518 goats and n = 165,518 sheep). Data was processed using the “EpiMuestra, version 1.0” computer package [27] with a 95% confidence interval, which is based on the following Formula (1):

(1) $$n=\frac{Z^{2}(P)(1-P)}{d^{2}}$$

where *n* is the sample size based on an infinite population, *P* is the expected prevalence, 50% was considered to maximize the sample size, *Z* = 1.96 confidence level value at 95%, and d is the absolute error.

The minimum required sample size was 381 per species. However, blood samples were collected from 389 goats and 385 sheep, totaling 774 animals.

Three to five mL of blood were collected from the jugular vein of each animal using vacuum tubes without anticoagulant. The samples were left to cloth for 3 h, centrifuged at 5000 rpm for 5 min, and 1 to 1.5 mL of serum was aliquoted into 1.5 mL microtubes and stored at −20 °C until the serological test for leptospirosis was performed. Of the 389 goat serum samples, 38.8% (151/389) were obtained from dairy production herds, while 61.2% (238/389) came from meat production herds. Regarding the management system, 46.0% (179/389) were from intensive farming (permanent confinement), 34.9% (136/389) were from semi-intensive (diurnal grazing paired with nocturnal confinement), and 19.0% (74/389) were from extensive farming (free ranging).

Of the 385 total sheep samples, 44.2% (170/385) were obtained from dairy production herds, while 55.8% (215/385) came from meat production herds. Based on the management system, 34.6% (133/385) were from intensive farming systems, 26.2% (101/385) were from semi-intensive systems, and 39.2% (151/385) were from extensive farming systems.

### 2.3. Serological Diagnosis

For the serological analysis of *Leptospira*, all samples were sent to and processed at the North Central Regional Laboratory (www.lcrn.mx, accessed on 6 March 2025), a SADER-accredited laboratory in the city of Guadalupe, Nuevo León, México. The microscopic agglutination test (MAT) with live antigens was performed following the recommendations of the World Organization for Animal Health (WOAH) Terrestrial Manual, which established the serological tests as the most widely used means for diagnosing leptospirosis and recognized it as the standard serological test [28]. A panel of 10 serovars corresponding to 8 different serogroups was used as an antigen to determine the presence of anti-*Leptospira* agglutinins (Table 1). A screening test was conducted at a 1:100 dilution against all serogroups. All samples showing agglutination ≥ 50% were considered positive and subjected to serial two-fold dilutions. The antigen with the highest titer was considered the infecting serogroup.

### 2.4. Epidemiological Survey

An epidemiological survey was conducted with all livestock owners, focusing on risk factors. Information was collected based on various variables, including production system (dairy production, meat production), management system (intensive; permanent confinement; semi-intensive; diurnal grazing paired with nocturnal confinement; and extensive; free ranging), number of animals per herd, coexistence with other domestic and wild animals, reproductive problems (abortions, stillbirths, mummifications, dystocia, low birth weight, congenital malformations, pregnancy rate), sanitary control, and veterinary assistance [21,22].

### 2.5. Statistical Analysis

The prevalence of positive animals was estimated based on the proportion of positive goats and sheep relative to the total number of sampled goats and sheep, with a 95% confidence interval (CI). For risk factor analysis, variables were separated into two categories: (1) Exposures (production system, management system, animals per herd, coexistence with other domestic and wild animals, sanitary control, veterinary assistance) and (2) infection-associated outcomes (reproductive problems); these were then analyzed in separated models. For both categories, risk factor analysis was conducted in two stages. A univariate analysis was performed using a chi-square test, where those variables demonstrating a *p*-value < 0.2 were selected for a second-stage analysis; for exposure variables, a stepwise logistic regression multivariate analysis with a significance level (SL) of 95% was conducted; meanwhile, for infection-associated outcomes, an odds–ratio analysis was performed. All statistical analyses were completed using SPSS software, version 25 (IBM, Armonk, NY, USA) [29].

## 3. Results

### 3.1. Serology

The overall prevalence was 13.6% (105/774). The seroprevalence in goats and sheep was 14.4% (56/389) and 12.7% (49/385), respectively (Table 2), with titers ranging from 1:100 to 1:400.

This study detected antibodies against all of the eight *Leptospira* serogroups, with most seroreactions presenting agglutinating titers of 1:100 (Table 3). Only six sera—three from each animal species—showed titers of 1:400. Four animals—two from each species—reacted to the Sejroe serogroup, one goat reacted to the Icterohaemorrhagiae serogroup, and one sheep reacted to the Bratislava serogroup. No titers above 1:400 were observed during testing (Table 3).

Among the seropositive group, the Sejroe serogroup exhibited the highest proportion of anti-*Leptospira* agglutinins, with frequencies of 60.7% (34/56) and 44.9% (22/49) in goats and sheep, respectively (Table 4).

Regarding production systems, meat production goats had a prevalence of 17.7% (42/238), while meat production sheep had a prevalence of 18.1% (39/215). In contrast, dairy production goats and dairy production sheep showed a prevalence of 9.3% (14/170) and 5.9% (10/170), respectively. At the management system level, goats had a prevalence of 16.2% (29/179), 11.8% (16/136), and 14.9% (11/74) under intensive, semi-intensive, and extensive farming systems, respectively. Conversely, sheep exhibited a prevalence of 14.7% (20/136), 9.9% (10/101), and 12.6% (19/151) under intensive, semi-intensive, and extensive farming systems, respectively.

### 3.2. Risk Factors

The results of the univariate analysis, including variables associated (*p <* 0.2) with the presence of anti-*Leptospira* agglutinins against one or more *Leptospira* serogroups in goats and sheep, are presented in Table 5. In both goat and sheep herds, the univariate analysis indicated that association with seropositivity to *Leptospira* was related to biosecurity deficiencies, herd management, and contact with domestic and wild animals.

Additionally, Table 6 presents the risk factors identified through multivariate logistic regression analysis. The identified risk factors in goats included lack of quarantine measures (OR:3.3), contact with wild pigs (OR:3.1), lack of veterinary assistance (OR:2.2), contact with domestic cattle (OR:2.1), and meat production (OR:2.1). Meanwhile, risk factors in sheep included contact with domestic cattle (OR:4.1), ≥100 sheep per pen (OR:3.3), contact with feral pigs (OR:3.1), meat production (OR:2.6), and lack of veterinary assistance (OR:2.3). Table 7 presents the association of clinical disorders and seropositivity against *Leptospira.*

## 4. Discussion

This study provides, for the first time in Mexico, important serological and epidemiological information on leptospirosis infections in small ruminants raised in semi-arid areas. The estimated average prevalence in small ruminants (13.57%) suggests natural infection and the circulation of different *Leptospira* spp. serogroups. However, further studies are needed to confirm whether it is indeed endemic. The seroprevalence found in small ruminants in this study was lower compared to tropical climate regions such as Brazil (82.9%) [30], New Zealand (57%) [31], Saint Kitts and Nevis (39.4%) [32], and Tunisia (25%) [33], but similar to other studies conducted in semi-arid climates such as Colombia (13.9%) [34], a semi-arid region of Brazil (13.3%) [35], and Iran (9.6%) [23]. At least 74.8% of all MAT-positive animals had antibody titers of 1:100 and 1:200. Other studies indicate that small ruminants tend to exhibit low serological titers against serogroups adapted to these animals [23,36]. The MAT test is considered the gold standard for diagnosing leptospirosis, as it allows the identification of the circulating serogroup in a region [28]. MAT, as a serological test, reveals the most frequent serogroup within and among herds, as well as the magnitude of antibody titers, which depend on the level of exposure to *Leptospira* in the studied population [2,23].

All small ruminant herd owners surveyed confirmed that they do not vaccinate their animals against leptospirosis, meaning that the low antibody titers (1:100 and 1:200) may indicate chronic infection. This is the first study exploring multiple *Leptospira* spp. serogroups in small ruminants in a semi-arid region of Mexico. This study identifies Sejroe as the most frequently adapted serogroup to small ruminants in the region, consistent with previous reports in Mexico [12,25]. It has been established that infections in small ruminants depend on coexistence with other animals, such as cattle. The presence of these animals in herds facilitates Sejroe infection in small ruminants. The second most common serogroup in goats was Icterohaemorrhagiae, while in sheep, it was Pomona. Although rats are considered the primary reservoirs of the Icterohaemorrhagiae serogroup, no association was found between rodent presence in herds and infections. Pigs are the main carriers of the Pomona serogroup. A study revealed a high seroprevalence of Pomona in feral pigs, suggesting that coexistence with these animals may favor infection with this serogroup [37].

According to the logistic regression model, small ruminants coexisting with domestic cattle had 4.08 times higher probability of testing positive. This finding contrasts with a study in small ruminants in Brazil, which reported a higher probability of infection in herds coexisting with dogs (OR:12.9) compared to those coexisting with cattle (OR:1.06) [38].

Additionally, herds with more than 100 animals were identified as a risk factor (OR:3.2) associated with seropositivity to any serogroup. It has been reported that as the number of animals per herd increases, so does the likelihood of exposure to *Leptospira*, resulting in a higher probability of testing positive [39]. Based on epidemiological survey data, a large proportion of the evaluated herds lacked technical assistance, meaning that the absence of veterinary medical assistance (OR:2.3) was identified as a risk factor associated with leptospirosis infections. This finding suggests a lack of awareness about leptospirosis prevention and control measures among farmers, who likely seek veterinary services only after the disease has become established within the herd. As for the clinical outcomes, it was established that reproductive disorders are closely linked to the presence of the infection within the herd. Although leptospirosis does not usually cause malformations, it has been seen that this bacterium can be involved with other infectious and non-infectious agents causing reproductive problems and congenital malformations such as *Chlamydia abortus* [40].

Leptospirosis in small ruminants caused by serogroups adapted to these animals generally results in subclinical infections that progress to chronic disease due to the persistence of the bacteria in the kidneys [36]. Chronically infected animals play a significant role in the epidemiology of the disease, contributing to the endemic nature of infection within herds, the environment, and the onset of reproductive problems in affected animals [23]. Congenital abnormalities, low birth weight, abortions, or ≤50% pregnancy rate, were the clinical impacts induced by *Leptospira*. However, although such infections go unnoticed or are not suspected, they lead to significant economic losses which are difficult to recognize [41], but a prevalence of 14.4% in goats means there are 59,547 diseased animals in a population of 413,518. If each animal costs $4000 MN, the losses will be $238.2 million pesos (US $11,910 billion).

It is crucial to emphasize that this is the first study associating risk factors with leptospirosis in small ruminants in a semi-arid region of Mexico. Under the One Health approach and considering the zoonotic and economic relevance of leptospirosis, local studies are necessary to better understand the epidemiology of the disease in small ruminants in these regions and to implement appropriate, regionally adapted prevention and control strategies [6,42].

## 5. Conclusions

This is the first study to determine the prevalence of leptospirosis in small ruminants in the semi-arid northeastern region of Mexico. The absence of veterinary assistance, coexistence with domestic cattle, and the presence of >100 animals per herd were associated with a high seroprevalence of *Leptospira*, particularly against the Sejroe serogroup. Furthermore, we conclude that there is a need for improved prophylactic assistance for disease prevention, as well as educational initiatives for farmers to implement biosecurity measures within their herds.

## Figures and Tables

**Table 1:** *Leptospira* strains evaluated as antigen in the serological MAT test.

Species	Serogroup	Serovar	Strain
*L. interrogans*	Sejroe	Hardjo	Hardjo-prajitno
	Sejroe	Wolffi	3707
	Pyrogenes	Pyrogenes	Salinem
	Pomona	Pomona	Pomona
	Australis	Bratislava	Jez-Bratislava
	Canicola	Canicola	Hond Utech IV
	Icterohaemorrhagiae	Icterohaemorrhagiae	RGA
*L. borgpetersenii*	Sejroe	Hardjo	Hardjo-bovis
	Tarassovi	Tarassovi	Pepereletsin
*L. kirschneri*	Grippotyphosa	Grippotyphosa	Moskva V

MAT: Microagglutination using live antigens.

**Table 2:** Prevalence of leptospirosis in seropositive small ruminants in Nuevo León, México.

Species	N° Animals		Prevalence (%)	95% CI
	**Sampled**	**Seropositive**		
Goats	389	56	14.4	10.9–17.9
Sheep	385	49	12.7	9.4–16.1
Total	774	105	13.6	11.2–15.9

CI: Confidence interval.

**Table 3:** Frequency of anti-*Leptospira* agglutinins according to the highest titer in goats and sheep in Nuevo León, México.

Serogroups	1:100		1:200		1:400		Total	
	Goats	Sheep	Goats	Sheep	Goats	Sheep	Goats	Sheep
Sejroe	23	13	9	7	2	2	34	22
Icterohemorragiae	7	6	0	0	1	0	8	6
Australis	5	4	1	0	0	1	6	5
Pomona	3	7	1	0	0	0	4	7
Pyrogenes	2	4	0	0	0	0	2	4
Canicola	2	2	0	0	0	0	2	2
Grippotyphosa	0	2	0	0	0	0	0	2
Tarassovi	0	1	0	0	0	0	0	1
Total	42	39	11	7	3	3	56	49

**Table 4:** Distribution of *Leptospira* serogroup reactivity among seropositive goats and sheep in Nuevo León, México.

Serogroup	Seroreactive			Prevalence %		
	Goats	Sheep	Total	Goats	Sheep	Total
Sejroe	34	22	56	60.7	44.9	53.3
Icterohaemorrhagiae	8	6	14	14.3	12.2	13.3
Australis	6	5	11	10.7	10.2	10.5
Pomona	4	7	11	7.1	14.3	10.5
Pyrogenes	2	4	6	3.6	8.2	5.7
Canicola	2	2	4	3.6	4.1	3.8
Grippotyphosa	0	2	2	0	4.1	1.9
Tarassovi	0	1	1	0	2.0	1.0

**Table 5:** Univariate analysis of leptospirosis risk factors in goats and sheep in Nuevo León, México.

Variable	Seropositive	Seronegative	*X* ^2^	*p*-Value
**Goats**				
Contact with wild pigs			29.96	<0.001 *
Yes	38	100		
No	18	233		
No quarantine measures			42.820	<0.001 *
Yes	54	165		
No	2	168		
No veterinary assistance			6.649	0.01 *
Yes	38	164		
No	18	169		
Contact with cattle			6.455	0.011 *
Yes	36	153		
No	20	180		
Low birth weight			5.05	0.019 *
Yes	40	182		
No	16	151		
Meat production			5.259	0.009 *
Yes	42	196		
No	14	137		
Contact with domestic dogs			4.315	0.038 *
Yes	42	196		
No	14	137		
Abortions			2.431	0.120
Yes	38	189		
No	18	144		
Contact with domestic pigs			1.086	0.297
Yes	16	119		
No	40	214		
**Sheep**				
≥100 animals per herd			21.425	<0.001 *
Yes	26	74		
No	23	262		
Contact with cattle			15.143	<0.001 *
Yes	40	175		
No	9	161		
Meat production			12.841	<0.001 *
Yes	39	176		
No	10	160		
Congenital abnormalities			15.964	<0.001 *
Yes	25	80		
No	24	256		
Contact with wild pigs			11.848	<0.001 *
Yes	26	96		
No	23	240		
≤60 animals per herd			5.111	0.024 *
Yes	12	139		
No	37	197		
No veterinary assistance			4.559	0.033 *
Yes	36	193		
No	13	143		
Contact with domestic dogs			3.288	0.070
Yes	27	139		
No	22	197		
Abortions			39.636	<0.001 *
Yes	25	129		
No	24	207		

* Statistically significant 95%.

**Table 6:** Risk factors associated with leptospirosis in small ruminants in Nuevo León, Mexico.

Risk Factor	RC	SE	*p*-Value	OR	95% CI
**Goat**					
No quarantine measures	1.202	0.335	<0.001	3.3	1.7–6.4
Contact with wild pigs	1.118	0.307	<0.001	3.1	1.7–5.6
No veterinary assistance	0.777	0.306	0.011	2.2	1.9–4.0
Contact with cattle	0.750	0.300	0.012	2.1	1.2–3.8
Meat production	0.740	0.328	0.024	2.1	1.1–4.0
**Sheep**					
Contact with cattle	1.408	0.385	<0.001	4.1	1.9–8.7
≥100 animals per herd	1.174	0.312	<0.001	3.3	1.8–6.0
Contact with wild pigs	1.136	0.312	<0.001	3.1	1.7–5.7
Meat production system	0.951	0.372	0.011	2.6	1.7–7.3
No veterinary assistance	0.838	0.350	0.017	2.3	1.2–5.6

RC: Regression coefficient, SE: Standard error, OR: Odds ratio, CI: Confidence interval.

**Table 7:** Associated clinical disorders with leptospirosis in small ruminants in Nuevo Leon, Mexico.

Clinical Disorder	OR	*p*-Value	95% CI
**Goat**			
Low birth weight	1.9	0.023	1.1–3.3
Abortions	1.7	0.045	1.0–2.9
**Sheep**			
Congenital abnormalities	2.8	0.003	1.4–5.6
Abortions	1.7	0.045	1.0–2.9

OR: Odds ratio, CI: Confidence interval.

## Data Availability

The datasets generated or analyzed during the present study are available on request from the corresponding author R.A.-R.
